# Gut microbiota in combination with blood metabolites reveals characteristics of the disease cluster of coronary artery disease and cognitive impairment: a Mendelian randomization study

**DOI:** 10.3389/fimmu.2023.1308002

**Published:** 2024-01-15

**Authors:** Shihan Xu, Yanfei Liu, Qing Wang, Fenglan Liu, Yanfang Xian, Fengqin Xu, Yue Liu

**Affiliations:** ^1^ The Second Department of Geriatrics, Xiyuan Hospital, China Academy of Chinese Medical Sciences, Beijing, China; ^2^ National Clinical Research Center for TCM Cardiology, Xiyuan Hospital, China Academy of Chinese Medical Sciences, Beijing, China; ^3^ Key Laboratory of Disease and Syndrome Integration Prevention and Treatment of Vascular Aging, Xiyuan Hospital of China Academy of Chinese Medical Sciences, Beijing, China; ^4^ School of Clinical Medicine, Guangdong Pharmaceutical University, Guangzhou, China; ^5^ School of Chinese Medicine, Faculty of Medicine, The Chinese University of Hong Kong, Hong Kong, Hong Kong SAR, China

**Keywords:** Mendelian randomization, gut microbiota, blood metabolites, disease cluster, coronary artery disease, cognitive impairment

## Abstract

**Background:**

The coexistence of coronary artery disease (CAD) and cognitive impairment has become a common clinical phenomenon. However, there is currently limited research on the etiology of this disease cluster, discovery of biomarkers, and identification of precise intervention targets.

**Methods:**

We explored the causal connections between gut microbiota, blood metabolites, and the disease cluster of CAD combined with cognitive impairment through two-sample Mendelian randomization (TSMR). Additionally, we determine the gut microbiota and blood metabolites with the strongest causal associations using Bayesian model averaging multivariate Mendelian randomization (MR-BMA) analysis. Furthermore, we will investigate the mediating role of blood metabolites through a two-step Mendelian randomization design.

**Results:**

We identified gut microbiota that had significant causal associations with cognitive impairment. Additionally, we also discovered blood metabolites that exhibited significant causal associations with both CAD and cognitive impairment. According to the MR-BMA results, the free cholesterol to total lipids ratio in large very low density lipoprotein (VLDL) was identified as the key blood metabolite significantly associated with CAD. Similarly, the cholesteryl esters to total lipids ratio in small VLDL emerged as the primary blood metabolite with a significant causal association with dementia with lewy bodies (DLB). For the two-step Mendelian randomization analysis, we identified blood metabolites that could potentially mediate the association between genus Butyricicoccus and CAD in the potential causal links.

**Conclusion:**

Our study utilized Mendelian randomization (MR) to identify the gut microbiota features and blood metabolites characteristics associated with the disease cluster of CAD combined with cognitive impairment. These findings will provide a meaningful reference for the identification of biomarkers for the disease cluster of CAD combined with cognitive impairment as well as the discovery of targets for intervention to address the problems in the clinic.

## 1 Introduction

Over the past few decades, remarkable advancements in medical technology coupled with refined strategies in public health practices have led to a remarkable upsurge in the global population’s life expectancy. However, this progress has also brought forth a consequential escalation in the prevalence of individuals with multimorbidity (1). Multimorbidity has emerged as a pervasive clinical phenomenon, imposing substantial challenges on both patient well-being and healthcare delivery, particularly among the elderly cohort. Notably, in the age group of 65 to 84 years, approximately two-thirds of individuals contend with the burden of multimorbidity, while this figure skyrockets to a staggering 80% among those aged 85 years and above. Within the context of multimorbidity in elderly patients, the co-occurrence of CAD and cognitive impairment is a notable disease cluster that cannot be overlooked (2). CAD, being one of the leading causes of mortality among elderly patients worldwide, continues to exhibit an incessant rise in its incidence risk (3). Cognitive impairment constitutes a comprehensive syndrome characterized by acquired and persistent cognitive function impairments, leading to a decline in daily life and occupational capacities as well as behavioral changes (4). The severity of such impairments can range from mild cognitive impairment to severe dementia. Epidemiological studies indicate a gradual increase in the population of elderly individuals affected by cognitive impairment, thereby presenting a significant challenge to global public health (5). Studies have shown that CAD leads to a 27% increased risk of future dementia, and that patients with CAD combined with cognitive impairment are more likely to have a major adverse cardiovascular event, which puts the patient’s health at greater risk than if they had one disease, however, cognitive decline was not detected in as many as 50% to 80% of cardiac patients with comorbid cognitive impairment, which may be related to the choice of different cognitive function assessment scales and the timing of cognitive function tests (6–8). Therefore, it is essential to explore the biomarkers of the disease cluster of CAD combined with cognitive impairment in order to construct more efficient and objective screening methods as well as to search for more possible therapeutic targets. With the rapid development of genomics, metabolomics and macro-genomics of gut flora, it gives us a great deal of opportunity to achieve the above mentioned goals.

A large body of evidence suggests that the gut microbiota plays a crucial role in the onset and progression of diseases such as metabolic disorders, neurodegenerative diseases and cardiovascular diseases (9). Several clinical studies have found significant alterations in gut microbiota in individuals with CAD and cognitive impairment (10, 11). Changes in the gut microbiota can mediate the development of CAD through mechanisms such as chronic inflammation, promotion of atherosclerosis, and promotion of thrombosis (12). Imbalances in the gut microbiota can lead to neuroinflammation, immune system dysregulation, accumulation of brain amyloid proteins and tau-like proteins, as well as impaired blood-brain barrier permeability, ultimately contributing to cognitive impairment (13). These findings all suggest that gut microbiota possess great potential as biomarkers and therapeutic targets for the disease cluster of CAD combined with cognitive impairment. However, it is worth noting that the changes in gut microbiota found in different current studies are not consistent, or even appear to be opposite, which may be related to participant selection bias and confounding by confounding factors, and these uncertainties create an obstacle to the specific clinical application of intestinal flora (14, 15). The human blood metabolome provides additional opportunities to identify multiple disease pathogenesis, improve multiple disease risk prediction, and explore multiple disease intervention targets through untargeted assessment of circulating small molecules (16). Study shows that both CAD patients and cognitively impaired patients display a broad set of blood metabolites disorders, these findings provide new directions for identifying biomarkers for the disease cluster of CAD combined with cognitive impairment and for exploring precise intervention targets (17, 18). In addition, studies have shown that blood metabolites mediate the effects of gut microbiota on CAD as well as cognitive impairment (18, 19). Therefore, combining blood metabolites with gut microbiota can be very helpful for the discovery of biomarkers and precise intervention targets for the disease cluster of CAD combined with cognitive impairment. However, specific clinical applications of blood metabolites also face large obstacles, as do gut microbiota, which arise mainly from risk factors associated with CAD and cognitive impairment such as hypertension, diabetes, and smoking, as well as interference from potential confounders. These confounding factors make it difficult to identify gut microbiota and blood metabolites that are causally associated with the disease clusters of CAD combined with cognitive impairment.

MR is an analytical method that utilizes genetic variation to simulate randomized controlled trials, enabling causal inference between risk factors and diseases. It can reduce the impact of confounding factors and reverse causation (20). The rapid growth of publicly available genome-wide association study (GWAS) data, the emergence of new methodologies, advancements in molecular epigenetics, and omics technologies have provided excellent opportunities for MR to explore causal relationships between complex diseases and other factors. Nowadays, large-scale GWAS data on gut microbiota, blood metabolites, CAD, and cognitive impairment are publicly available, creating opportunities for us to investigate the causal associations between gut microbiota, blood metabolites, and the disease cluster of CAD combined with cognitive impairment through MR. Previously, some researchers have found causal links between specific gut microbiota and CAD and Alzheimer’s disease (AD) by MR, and others have explored causal links between blood metabolites and AD by MR (21–23), but they have not gone further to explore the causal links between the gut microbiota and the blood metabolites in patients with CAD as well as AD, and they have not focused their attention on the disease cluster of CAD combined with cognitive impairment. Therefore, we will conduct a more comprehensive and in-depth exploration by MR to provide new insights into biomarkers and treatments for the disease cluster of CAD combined with cognitive impairment.

In this study, we aim to explore the causal relationships between gut microbiota, blood metabolites, and the disease cluster of CAD combined with cognitive impairment using TSMR. Additionally, we will employ MR-BMA analysis to identify the gut microbiota and blood metabolites with the strongest causal associations. Furthermore, we will investigate the mediating role of blood metabolites through a Two-Step Mendelian Randomization design.

## 2 Methods

### 2.1 Study design

**Figure 1:**
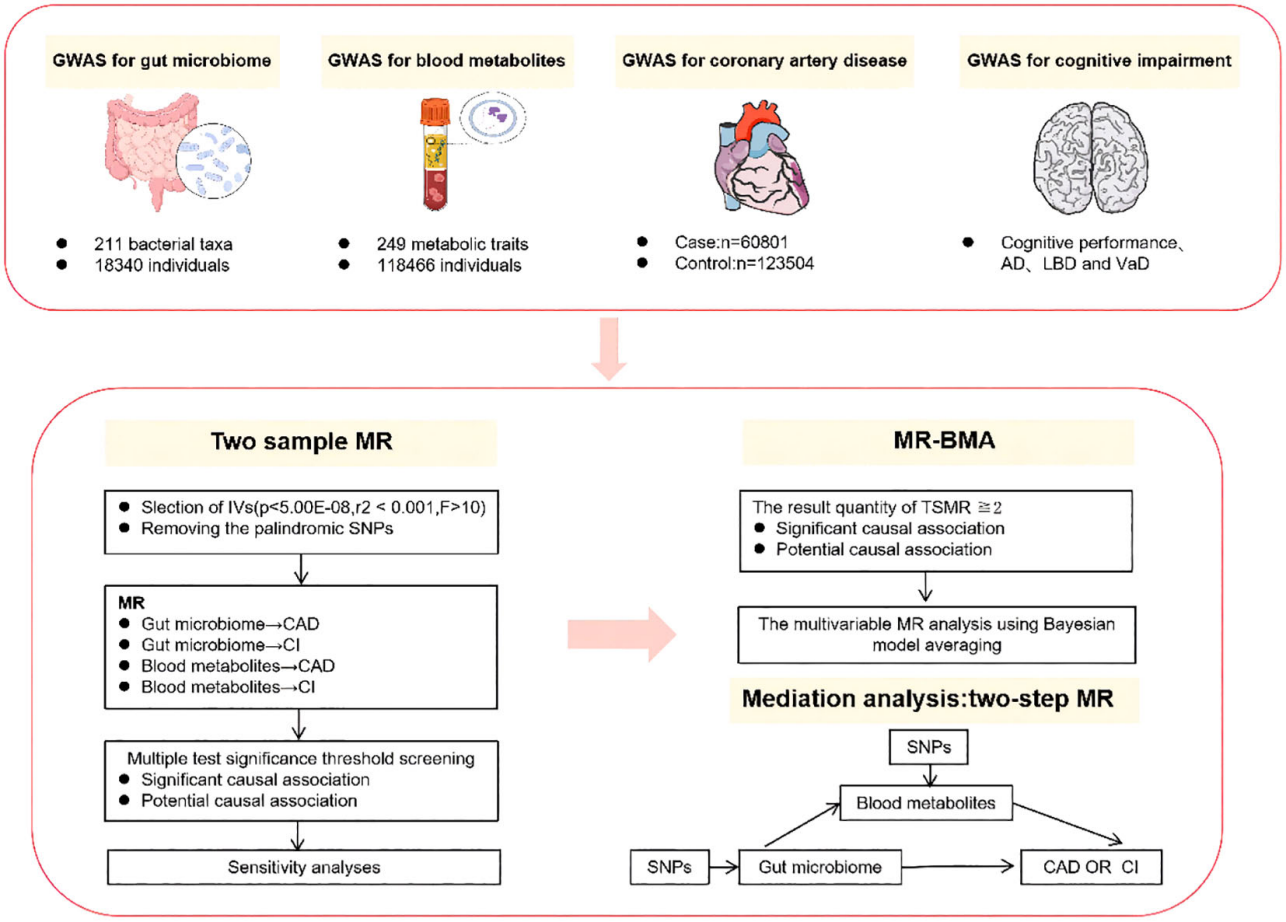
Summary of analyses performed in this study. In this study, the cognitive impairment phenotype includes cognitive performance as well as specific subtypes of cognitive impairment, namely AD, DLB, and VaD. Only the results of the two samples MR that have been selected through sensitivity analysis will undergo subsequent MR-BMA and mediation analysis. AD, alzheimer’s disease; DLB, dementia with lewy body; VaD, vascular dementia. MR-BMA, Bayesian Model Averaging Multivariable Mendelian Randomization.

The study design is depicted in **Figure 1**. We first conducted TSMR analyses for the following causal associations (1): gut microbiota and CAD (2), gut microbiota and cognitive impairment (3), blood metabolites and CAD, and (4) blood metabolites and cognitive impairment. Multiple testing significance thresholds were applied to assess the associations between gut microbiota and blood metabolites, resulting in significant causal associations and potential causal associations. Subsequently, we performed MR-BMA analyses on the TSMR results that met the criteria: the result qunanitity of TSMR≧2 and passed sensitivity analysis for potential causal associations and significant causal associations. Furthermore, for both potential causal associations and significant causal associations, we employed a Two-Step MR design to explore the mediating effects of blood metabolites in the influence of gut microbiota on CAD and cognitive impairment.

### 2.2 Data source

The GWAS data on gut microbiota used in this study were obtained from the international consortium MiBioGen. This consortium analyzed the genetic typing data of 18,340 individuals from 24 cohorts, as well as the 16S rRNA gene sequencing profiles of fecal microbiota (24). This is a large-scale, multi-ethnic, whole-genome meta-analysis study. For this study, we limited our analysis to individuals of European ancestry, and a total of 211 taxonomic units of gut microbiota were included in the analysis(131 genera, 35 families, 20orders, 16 classes, and 9 phyla). The GWAS data for blood metabolites was obtained from a large metabolomics dataset recently released by the UK Biobank (25). Researchers utilized high-throughput nuclear magnetic resonance spectroscopy (Nightingale Health Plc; Biomarker quantification version 2020) to measure non-fasting EDTA plasma samples from a randomly selected subset of 118,466 individuals from the UK Biobank. A total of 249 metabolic traits were quantified in this measurement, including 168 in absolute levels and 81 in ratio measures. These traits encompassed various subclasses of lipoprotein lipids, fatty acids, and their compositions, as well as numerous low-molecular-weight metabolites such as amino acids, ketone bodies, and glycolytic metabolites quantified in molar concentrations. The GWAS data for CAD is derived from a large-scale, multi-ancestry meta-analysis based on the 1000 Genomes Project (26). The meta-analysis aggregated data from 48 studies, encompassing 60,801 CAD cases and 123,504 controls. Among the participants, 77% had European ancestry. The included cases consisted of individuals with coronary artery stenosis >50%, chronic stable angina, acute coronary syndrome, or myocardial infarction. The degree of cognitive impairment can range from mild cognitive impairment to severe dementia. In elderly patients, this syndrome is often manifested in conditions with higher prevalence, such as AD, DLB, and vascular dementia (VaD). Therefore, in this study, the cognitive impairment phenotype includes general cognitive function as well as specific subtypes of cognitive impairment, namely AD, DLB, and VaD. The GWAS data for cognitive performance is sourced from a weighted meta-analysis conducted by the Social Science Genetic Association Consortium (SSGAC) (27). This analysis includes studies on cognitive function from the COGENT Consortium and the UK Biobank (UKB), comprising a total of 257,841 individuals. The COGENT Consortium’s sub-studies (n=35) involved participants who underwent neurocognitive testing in an average of eight sessions, with at least three cognitive domains assessed. Principal component analysis was performed on the test scores, and the first unrotated principal component was extracted to assess participants’ cognitive performance. In the UK Biobank, participants were required to complete a language-based numerical reasoning test consisting of 13 logical and reasoning questions within two minutes to evaluate cognitive performance. The GWAS data for AD is sourced from the International Genomics of Alzheimer’s Project (IGAP) (28). IGAP provides a meta-analysis of GWAS based on discovery samples from four consortia and the IGAP meta-analysis includes 21,982 cases diagnosed with late-onset AD and 41,944 cognitively normal controls. The GWAS data for DLB is derived from a genome-wide association study (29). This study included a total of 6,618 participants of European ancestry, consisting of 2,591 DLB cases and 4,027 neurologically healthy controls. The individuals included in the case group were diagnosed as pathologically confirmed or clinically probable DLB based on consensus criteria (30). The selection of all control group participants was based on the absence of evidence of cognitive decline in their clinical history and the absence of abnormalities in neurological examinations. The GWAS data for VaD is derived from FinnGen, an ongoing research project. This project combines genetic data from the Finnish Biobank with healthcare data collected and processed by the National Institute for Health and Welfare in Finland. As of now, the project involves a substantial number of individuals, reaching up to 377,277 participants (31). We have obtained the GWAS data for VaD from the R5 dataset released by FinnGen. The dataset consists of 881 VaD cases and 211,508 controls. VaD primarily refers to the condition defined by the diagnostic codes of the International Classification of Diseases, 10th edition (ICD-10) and ICD-9 (ICD-10: F01, ICD-9: 4378).

### 2.3 Genetic instrumental variable selection

The core component of Mendelian randomization studies involves utilizing single nucleotide polymorphisms (SNPs) as instrumental variables (IVs). SNPs, serving as IVs, can overcome confounding factors inherent in observational research, provided that we obtain effective IVs through stringent selection criteria. Firstly, we employed a threshold of P <1×10^-5^ to select SNPs associated with the gut microbiota. These SNPs were utilized as genetic instrumental variables, following the approach used in previous gut microbiota MR studies, P<1×10^-5^ represents the optimal threshold for selecting genetic predictive factors associated with gut microbial characteristics, as demonstrated in previous studies (32). For blood metabolites, P <5×10^-8^ represents the threshold for selecting genetic predictive factors. Secondly, we computed the F-statistic for each genetic instrumental variable and selected IVs with F>10. Thirdly, to minimize bias introduced by linkage disequilibrium (LD), we clumped all SNPs based on a LD threshold of r^2^<0.001 within a distance of ± 10,000 kb. This clumping process was performed using the 1000 Genomes European reference panel separately, limited to SNPs with minor allele frequency>0.01. Finally, we ensured that the impact of a SNP on a specific outcome and exposure is adjusted to the same allele, ensuring allele-specific harmonization. Additionally, we excluded palindromic SNPs from the analysis.

### 2.4 Statistical analyses

We conducted TSMR analyses to assess the causal effects of the following associations (1): gut microbiota and CAD (2), gut microbiota and cognitive impairment (3), blood metabolites and CAD, and (4) blood metabolites and cognitive impairment. For exposures with multiple IVs, the inverse-variance weighted (IVW) method with multiplicative random effects is regarded as the most efficient approach for obtaining causal effect estimates, and this method can also account for heterogeneity in causal estimates (33). Therefore, we selected the IVW method with multiplicative random effects as the primary analysis approach for MR. For exposures with only a single IV, we utilized the wald ratio analysis method to estimate the causal effects. Furthermore, following previous studies (34), we established multiple testing significance thresholds for gut microbiota and blood metabolites. For each level of gut microbiota feature (phylum, class, order, family, genus), the threshold was defined as P <0.05/n, where n represents the number of bacteria included with valid instrumental variables at the corresponding feature level. For different categories of blood metabolites, the threshold was defined as P <0.05/n, where n represents the number of metabolites included with valid instrumental variables in the respective category. After applying the multiple testing significance threshold, the results that met the threshold were considered as significant causal associations. On the other hand, results that did not pass the threshold but had a p-value less than 0.05 were considered as potential causal associations. These potential associations indicate a trend towards a causal relationship, further investigation is needed to confirm their significance. We further assess the robustness of both significant causal associations and potential causal associations using MR Egger regression (35), Weighted Median (36), Weighted Mode (37), and the MR Pleiotropy Residual Sum and Outlier (MR-PRESSO) (38). The reason for choosing these methods is that causal inference under different assumptions can better help us detect whether there is a violation of the MR assumptions (39). In addition to the robust MR methods mentioned above, we conducted a series of other sensitivity analyses. Firstly, we assessed heterogeneity in causal inference by calculating Cochran’s Q statistic (40). Additionally, we evaluated whether the estimation of causal effects was influenced by specific variants through leave-one-out analyses (41). Furthermore, we utilized the MR Steiger directionality test to determine the direction of causality between the exposure and outcome. In cases where the Steiger test identified stronger associations between certain genetic instrumental variables and the outcome, we removed these variants and performed the analysis again (42).

The gut microbiota and blood metabolites exhibit strong correlations, both in terms of their phenotypic characteristics and genetic variations. This phenomenon has been demonstrated in previous MR analyses (23), which also explains the significant overlap of IVs in TSMR analysis. While TSMR can infer the causal effects of individual exposures, it cannot exclude the possibility of non-independent factors that may result in correlated exposure groups acting on the outcome together. Therefore, we employed MR-BMA to investigate the causal effects of the exposure on the outcome by reducing potential biases that could arise from the aforementioned factors. This method enables the modeling of multiple correlated risk factors together and identifies the true causal risk factors, unlike traditional multivariable MR, this approach is particularly suitable for high-throughput and highly correlated data (43).

We performed further analysis on the following TSMR results using MR-BMA: the result qunanitity of TSMR≧2 and passed sensitivity analysis for potential causal associations and significant causal associations. We conducted MR analysis on multiple exposure combinations using a weighted linear regression model similar to the IVW method. In the Bayesian framework, we evaluated the posterior probability (PP) of specific models and calculated the marginal inclusion probabilities (MIP) for each exposure by summing the PP of each exposure included in all models. The exposures were ranked in descending order based on their MIP, with the exposure having the highest MIP considered to have the strongest causal association with the outcome. Additionally, we calculated the model-average causal effect (MACE), which reflects the average direct effect of each exposure on the outcome, independent of any other exposures included in the model. Q statistics and Cook’s distance were also calculated to identify outlying variables and influential points in the model. After excluding these outliers, we repeated the above operations. Finally, we employed a two-step MR analysis design to determine the mediating role of blood metabolites in the causal relationship between gut microbiota and diseases, separately for significant causal associations and potential causal associations. We calculated the proportion of mediation by blood metabolites using the indirect effect divided by the total effect (β1 × β2/β3), where β1 represents the effect of gut microbiota on blood metabolites, β2 represents the effect of blood metabolites on the outcome, and β3 represents the effect of gut microbiota on the outcome. All β values were obtained through TSMR analysis.

All the analyses were conducted on the R platform (version 4.2.1). The “TwoSampleMR” and “ggplot2” packages were used for statistical analysis and data visualization. The MR-BMA analysis was performed based on the R-code available on GitHub (https://github.com/verena-zuber/demo_AMD). All GWAS summary data was obtained from the IEU-OpenGWAS platform, and the MRC IEU UK Biobank GWAS pipeline was used to generate the data (44).

## 3 Result

### 3.1 Instrument variables included in analysis

We extracted valid IVs from gut microbiome and blood metabolites GWAS based on the aforementioned selection criteria. The detailed characteristics of these IVs can be found in **Supplementary Tables 1, 2**. All SNPs used for analysis have an F-statistic greater than 10.

### 3.2 Two-sample MR analysis

**Figure 2:**
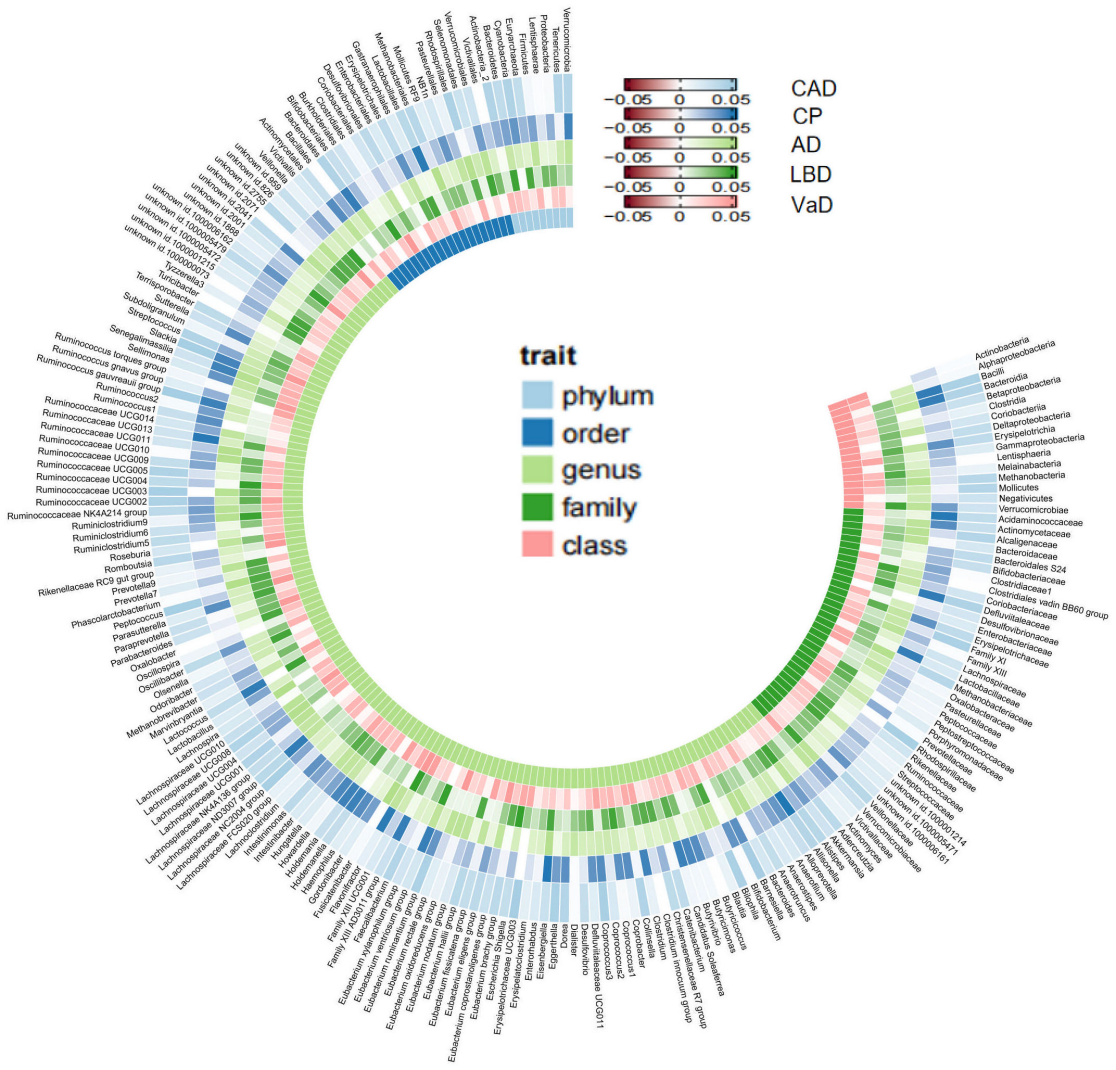
All results of gut microbiome. Our study is primarily based on IVW as the main analysis method. Therefore, this figure displays the p-values of IVW. The five concentric heatmaps from outer to inner represent the IVW analysis results of the gut microbiota with respect to CAD, cognitive performance, AD, LBD, and VaD, respectively. The lighter the color, the more significant the results. The innermost circle heatmap represents 211 taxonomic units of gut microbiota and the taxa to which they belong.

**Figure 3:**
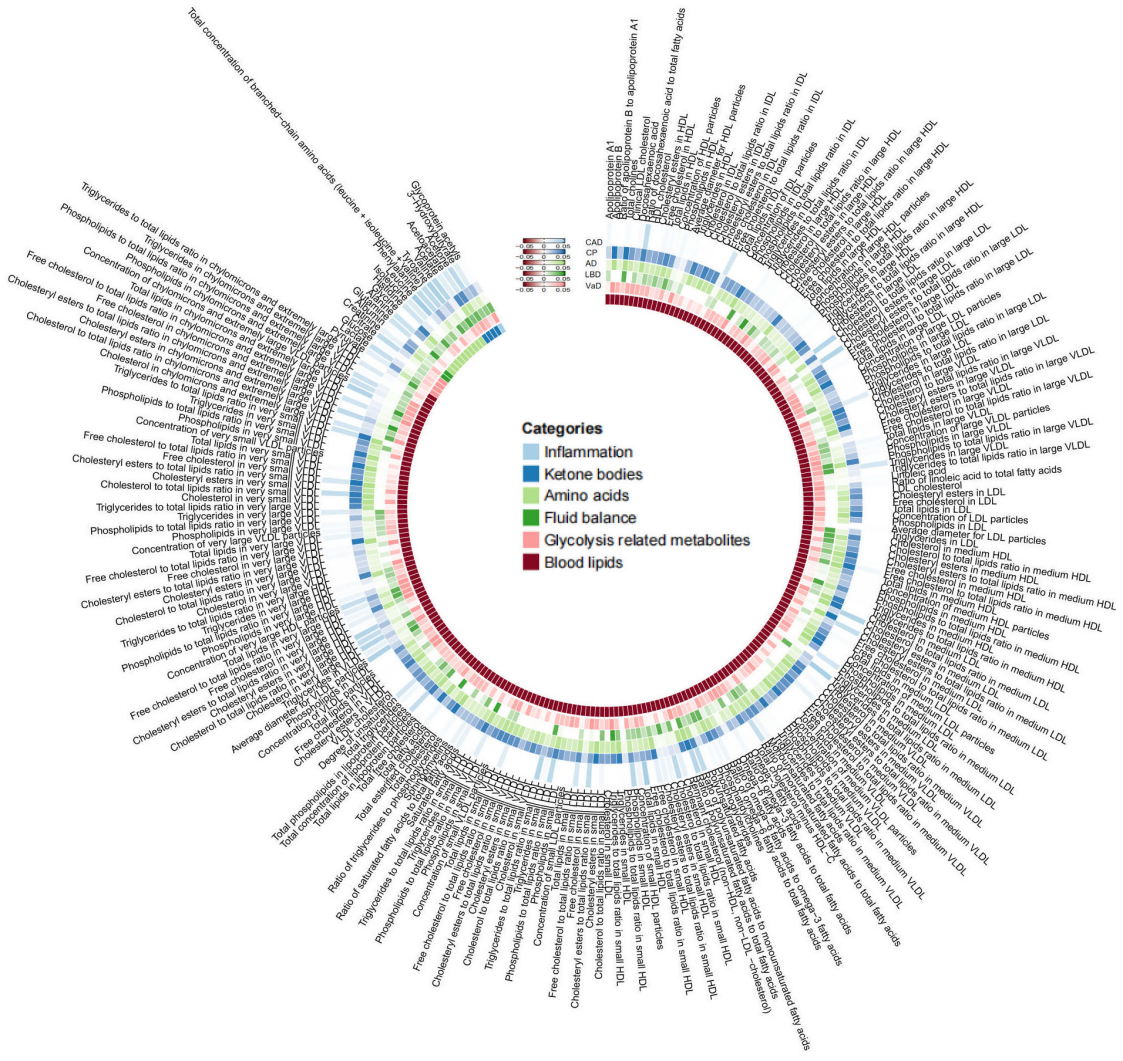
|All results of blood metabolites. This figure displays the p-values of IVW. The five concentric heatmaps from outer to inner represent the IVW analysis results of the blood metabolites with respect to CAD, cognitive performance, AD, DLB, and VaD, respectively. The lighter the color, the more significant the results. The innermost circle heatmap represents 249 taxonomic units of blood metabolite and the taxa to which they belong.

As mentioned earlier, we established multiple testing significance thresholds for each level of gut microbiota features and blood metabolites categories. The multiple testing significance thresholds for gut microbiota features are as follows: phylum *P*=0.0056 (0.05/9), class *P*=0.0031 (0.05/16), order *P*=0.0025 (0.05/20), family *P*=0.0015 (0.05/34), genus *P*=0.0004 (0.05/131). The multiple testing significance thresholds for blood metabolites categories are as follows: blood lipids *P*=0.0002 (0.05/228), amino acids *P*=0.005 (0.05/10), glycolysis-related metabolites *P*=0.0125 (0.05/4), ketone bodies *P*=0.0125 (0.05/4), fluid balance *P*=0.025 (0.05/2), inflammation *P*=0.05 (0.05/1). All results of TSMR for gut microbiota are shown in the **Figure 2** and all results of TSMR for blood metabolites are shown in the **Figure 3**.

#### 3.2.1 Significant causal association

In the MR analysis of gut microbiota and CAD, we did not find any gut microbiota that showed significant causal associations after applying the multiple testing significance threshold. However, in the MR analysis of cognitive impairment, we identified gut microbiota with significant causal associations, as shown in **Table 1**. In the MR analysis of blood metabolites and CAD, after applying the multiple testing significance threshold, we discovered 54 blood metabolites that showed significant causal associations with CAD. Among them, the most significant one was the ratio of apolipoprotein B to apolipoprotein A1 (OR=1.7369, 95% CI=1.5908 to 1.8964, P=7.34×10^-35^, IVW). In the MR analysis of blood metabolites and AD, glutamine (OR=0.8383, 95% CI=0.7605 to 0.9242, P=3.91×10^-4^, IVW) was identified as a blood metabolite significantly associated with AD. In the MR analysis of blood metabolites and DLB, we found 14 blood metabolites that were significantly associated with DLB. Among them, the most significant one was concentration of intermediate-density lipoprotein (IDL) particles (OR=1.5301, 95% CI=1.2631 to 1.8536, P=1.38×10^-5^, IVW). In the MR analysis of blood metabolites and other cognitive impairment phenotype, after applying the multiple testing significance threshold, we did not find any blood metabolites that showed significant causal associations. For more detailed information regarding the significant causal associations of gut microbiota and blood metabolites, you can refer to **Supplementary Table 3.**

**Table 1:** The significant causal associations in gut microbiota MR analysis.

Exposure	Outcome	Method	P	OR or beta	95%CI
Genus Roseburia	Cognitive performance	IVW	3.55×10^-5^	-0.07	-0.1 to -0.04
		MR Egger	0.86	0.008	-0.076 to 0.091
		Weighted median	0.003	-0.05	-0.09 to -0.006
		Weighted mode	0.17	-0.05	-0.11 to 0.01
Family Desulfovibrionaceae	AD	IVW	8.52×10^-4^	1.31	1.17 to 1.53
		MR Egger	0.21	1.36	0.88 to 2.1
		Weighted median	0.04	1.27	1.02 to 1.58
		Weighted mode	0.19	1.25	0.92 to 1.72
Order Desulfovibrionales	AD	IVW	1.62×10^-3^	1.27	1.09 to 1.47
		MR Egger	0.14	1.41	0.92 to 2.15
		Weighted median	8.8×10^-3^	1.32	1.07 to 1.62
		Weighted mode	0.14	1.27	0.94 to 1.72
Class Alphaproteobacteria	LBD	IVW	8.99×10^-4^	1.97	1.32 to 2.94
		MR Egger	0.43	1.93	0.43 to 8.78
		Weighted median	3.51×10^-3^	2.14	1.28 to 3.57
		Weighted mode	0.07	2.24	1.09 to 4.61
Class Deltaproteobacteria	VaD	IVW	2.39×10^-3^	12.28	2.43 to 61.93
		MR Egger	0.31	99.39	0.02 to 431040
		Weighted median	0.02	13.37	1.54 to 115.88
		Weighted mode	0.13	16.98	0.57 to 503.34

It should be noted that the direction of causal association between Genus Roseburia and cognitive performance has changed in the MR-Egger analysis. Therefore, we need to approach this result with greater caution.

#### 3.2.2 Potential causal association

In TSMR, we have identified multiple gut microbiota that have a potential causal association with CAD and cognitive impairment. Similar findings have also been observed in the analysis of blood metabolites. Further details regarding the potential causal associations can be found in **Supplementary Table 4**.

### 3.3 Sensitivity analyses

When reanalyzing the causal associations using MR Egger, weighted median, and weighted mode, most of the results showed consistent directions with the IVW method. However, there were also cases of inconsistent directions (**Supplementary Tables 3, 4**). In subsequent studies, we removed the potentially causal associations with inconsistent directions but retained those with significant causal associations despite the inconsistency, and we approached these results cautiously in further analyses. We did not find evidence of horizontal pleiotropy in the causal associations based on the intercept from MR-Egger analysis. The Cochran’s Q statistic showed heterogeneity, but as mentioned earlier, the inverse variance-weighted method with multiplicative random effects can account for the heterogeneity in the causal estimates (**Supplementary Table 5**). The leave-one-out analysis identified causal associations that are susceptible to specific SNP influences (**Supplementary Table 6**). After removing these causal associations, we performed MR-PRESSO analysis on the remaining causal associations, where we identified the presence of horizontal pleiotropic outlier variants. With the exception of the ratio of omega-6 fatty acids to omega-3 fatty acids on CAD, the remaining causal inferences remained consistent before and after removing the outliers (**Supplementary Table 7**). Following the aforementioned sensitivity analysis, we finalized a set of causal associations that exhibited more robust results (**Supplementary Table 8**), and these causal associations were subjected to further analysis.

### 3.4 Bayesian model averaging MR

After conducting the aforementioned sensitivity analysis, we performed MR-BMA analysis on the significant causal associations and potential causal associations that exhibited robust results. Additionally, we calculated Q statistics and Cook’s distance to identify outliers and influential points in the models. After excluding these outliers, we conducted MR-BMA analysis again, and the final results are presented in **Table 2**. For more detailed results, please refer to **Supplementary Tables 9, 10**.

**Table 2:** The result of MR-BMA analysis of significant and potential causal associations.

	Outcome	The strongest causal candidates	MIP	MACE
Significant causal association	AD	Order Desulfovibrionales	0.527	0.134
	CAD	Free cholesterol to total lipids ratio in large VLDL	0.318	0.107
	LBD	Cholesteryl esters to total lipids ratio in small VLDL	0.291	0.077
Potential causal association	CAD	Genus Oxalobacter	0.605	0.043
	CP	Genus Ruminococcaceae UCG003	0.838	0.037
	AD	Class Deltaproteobacteria	0.91	0.212
	LBD	Genus Ruminococcus gnavus group	0.906	-0.379
	VD	Family Bifidobacteriaceae	0.54	0.34
	CAD	Cholesterol to total lipids ratio in small VLDL	0.731	0.502
	LBD	Cholesteryl esters in VLDL	0.355	0.078

We performed MR-BMA analysis separately on the significant causal associations and potential causal associations obtained from TSMR analysis. In each MR-BMA analysis, we ranked the gut microbiota or blood metabolites based on the marginal inclusion probabilities derived from model diagnostics. The prior probability used in the MR-BMA analysis was set to 0.1, and the prior variance was set to 0.5.

### 3.5 Two-step MR

We conducted two-step MR analysis separately on the robust significant causal associations and the robust potential causal associations. However, in the significant causal associations, we did not find any intermediate blood metabolites that mediate the causal links between gut microbiota and CAD or cognitive impairment. In the potential causal associations, we discovered that omega-6 fatty acids (mediated proportion 38.2%), polyunsaturated fatty acids (mediated proportion 32.6%), sphingomyelins (mediated proportion 51.4%), and total phospholipids in lipoprotein particles (mediated proportion 43.3%) mediate the potential causal relationship between the genus *Butyricicoccus* and CAD. The results are illustrated in **Supplementary Table 11**.

## 4 Discussion

In this study, we conducted comprehensive MR analysis using large-scale GWAS summary data to investigate the causal relationships between gut microbiota, blood metabolites, and the disease cluster of CAD combined with cognitive impairment. The gut microbiota we found significant causal associations with cognitive impairment included Genus Roseburia, Family Desulfovibrionaceae, Order Desulfovibrionales, Class Alphaproteobacteria and Class Deltaproteobacteria. But unfortunately, in our study, we did not find gut microbiota that had significant causal associations with CAD. For blood metabolites, we found significant or potentially causal associations between a higher number of lipid metabolites and CAD as well as cognitive impairment, according to the MR-BMA results, the free cholesterol to total lipids ratio in large VLDL was identified as the key blood metabolite significantly associated with CAD. Similarly, the cholesteryl esters to total lipids ratio in small VLDL emerged as the primary blood metabolite with a significant causal association with DLB according to the MR-BMA results. We also found a significant causal association between glutamine and AD. And we were unable to find blood metabolites that acted as mediators in the significant causal associations through the two-step MR analysis. Overall, our findings provide new insights into potential biomarkers and precise therapeutic targets for studying the disease cluster of CAD combined with cognitive impairment.

Previous clinical studies have identified a correlation between gut microbiota and cognitive impairment, and our study further identifies a causal link between specific gut microbiota and cognitive impairment, this discovery will allow the gut microbiota to be better utilized in the identification of biomarkers for disease clusters as well as the discovery of therapeutic targets. In our study, family Desulfovibrionaceae as well as order Desulfovibrionales were significantly and causally associated with increased risk of AD, and previous clinical studies have corroborated our findings. The study by Hou et al (45) found higher abundance of family Desulfovibrionaceae and order Desulfovibrionales in AD patients, the same results were found in patients with cognitive impairment in the study by Park et al (46). Haran et al (47) combined with machine learning found that a representative species of the sulfate-reducing Desulfovibrio genus (D. fairfieldensis) could serve as a highly significant predictor of AD. The type genus of family Desulfovibrionaceae and order Desulfovibrionales is the Desulfovibrio genus, which is a Gram-negative sulphate-reducing bacterium that is widely distributed in the human intestinal tract and whose main metabolite is hydrogen sulphide (H_2_S), H_2_S as a gaseous transmitter plays a wide range of roles in the pathophysiology of the nervous system (48). Elevated blood levels of H_2_S and its metabolites have been found in AD patients, this alteration can make the blood-brain barrier(BBB) dysfunctional and lead to excitotoxic stress and cognitive impairment (49). Significant causal associations between genus Roseburia and cognitive performance were also found in our study, the relationship between intestinal flora and amyloid in cerebrospinal fluid was revealed by Verhaar et al. (50), who found that a decrease in the abundance of Roseburia hominis was associated with an increased chance of a positive cerebrospinal fluid amyloid test. Haran et al (47) also found a reduction in Roseburia hominis abundance in elderly patients with AD. Genus Roseburia is an anaerobic, Gram-positive bacterium whose main metabolite is short-chain fatty acids (SCFA) (51), SCFA can enter the CNS through active or passive pathways and have a wide range of effects on neurotransmitters, mitochondrial function, neuroimmunomodulation, and related gene expression, as SCFA accumulates in cells, it gradually leads to intracellular acidification and affects neuronal function subconsciously by altering calcium signaling and neurotransmitter release (52). Significant causal association between class Alphaproteobacteria and DLB and significant causality between class Deltaproteobacteria and VaD were also found in our study. Previous studies have found that the abundance of phylum Proteobacteria as well as order Deltaproteobacteria is elevated in AD patients and that Alphaproteobacteria correlates with mood disorders in AD patients (45, 53). Although current clinical studies have identified this relationship, the underlying pathophysiological processes are still unclear and more research is still needed to further explore the underlying mechanisms. Among CAD patients, genus Roseburia, family Desulfovibrionaceae, and order Desulfovibrionales have been directly or indirectly associated with CAD (54, 55). At the same time, the main metabolites of these intestinal flora, H_2_S and SCFA, also influence the pathophysiological processes of CAD (56, 57). Although no causal associations between these gut microbiota and CAD were found in the present study, this may be due to the high degree of heterogeneity of gut microbiota among populations, which makes MR analyses insufficient to detect potential causal associations between all gut microbiota and all phenotypes, in the case of CAD, these alterations in gut microbiota cannot be ignored, and when these gut microbiota are dysregulated, it may indicate that patients with CAD have a concomitant and unrecognized decline in cognitive function or an elevated risk of future co-morbid cognitive impairment. Interventions with these gut microbiota may also contribute to the improvement of the disease cluster of CAD combined with cognitive impairment.

By analyzing the causal links between blood metabolites and CAD and cognitive impairment, we found that lipids play an important role in the development of CAD and cognitive impairment. Of the lipid metabolites causally associated with CAD, the strongest causal association was Free cholesterol to total lipids ratio in large VLDL. Elevated VLDL cholesterol levels increase the risk of CAD by 2.19-3.36-fold in people with low-density lipoprotein (LDL) cholesterol in the normal range and in the absence of other major risk factors (58). VLDL cholesterol can explain the increased risk of myocardial infarction associated with elevated levels of apolipoprotein B (apoB), while VLDL triglycerides do not contribute to this risk (59). VLDL uptake by macrophages promotes a shift in their inflammatory phenotype, increases phagocytosis, and promotes foam cell formation, in addition, VLDL increases cholesterol deposition in atherosclerotic plaques, and cholesterol crystals are a typical feature of atherosclerotic plaque necrosis, its increase leads to plaque instability as well as a tendency to rupture (60). In our study, lipid metabolites were also significantly and causally associated with LBD, and the cholesteryl ester to total lipid ratio in small VLDL showed a stronger causality than other metabolites. The main pathological feature of DLB is lewy bodies (LB), which are enriched not only in α-synuclein (α-syn) but also in lipids (61). α-Syn stimulates microglia activation, which induces reactive oxygen species production and leads to apoptosis of pericytes, and the reduction of pericytes leads to a dysfunction of the blood-brain barrier (BBB), which allows for the entry of VLDL into the brain (62, 63). The N-terminal region of α-syn interacts with apolipoproteins to increase their tendency to aggregate, and cholesterol also regulates the expression and aggregation of α-syn, leading to neuronal cell dysfunction and death (64). Potential causal associations between many lipid metabolites and AD as well as VaD were similarly identified in our study, which is consistent with the large number of clinical studies that have found a correlation between lipid alterations and AD and VaD (65–67). Lipid abnormalities may contribute to the development of cognitive impairment through impaired BBB function, neuroinflammation, and cerebrovascular dysfunction (68). Therefore, it is not difficult to find that lipid metabolites are non-negligible factors in the development of cognitive impairment, and the causal association between them and the mechanisms behind them still need to be investigated more thoroughly. In conclusion, the impact of lipids, especially VLDL, on the disease cluster of CAD and cognitive impairment is very important, and more precise studies on biomarkers and therapeutic targets in the disease cluster of CAD and cognitive impairment is very important may be possible in the future through lipidomics. In addition to lipids, we also found a significant causal association between Gln and AD. Gln, the most abundant and versatile amino acid in human blood, exerts neuroprotective effects by buffering the increase of reactive oxygen species and reducing the damage caused by oxidative stress (69, 70). Furthermore, dysregulation of the glutamate-glutamine cycle may lead to neuronal death mediated by glutamate (71). Gln is likewise closely related to CAD, Gln is independently associated with the onset and severity of CAD (72), and Gln has the potential to alleviate various cardiovascular risk factors such as hypertension, hyperlipidemia, and diabetes (73). Therefore, blood Gln could also be one of the biomarker candidates for the disease cluster of CAD combined with cognitive impairment. Screening for biomarkers in the disease cluster of CAD combined with cognitive impairment is hampered by observational studies, mainly due to biases in participant selection, as well as inconsistencies in sample sizes, sequencing protocols, bioinformatics processes and statistical analysis methods, all of which may lead to inconsistencies in the results (74). And our study may be able to alleviate this hindrance by incorporating MR analysis.

To the best of our knowledge, this is the first MR analysis conducted on the gut microbiota and blood metabolites of the disease cluster. Our study will provide a meaningful reference for the discovery of biomarkers and targets for intervention in the disease cluster of CAD combined with cognitive impairment to address the problems in the clinic. Meanwhile, our study can also provide more ideas and directions for understanding the mechanisms behind the disease cluster of CAD combined with cognitive impairment. However, our study has some limitations. Firstly, although we utilized results from a large-scale blood metabolomics analysis in the UK Biobank, the included metabolites are not comprehensive. Subsequent GWAS analysis should be conducted on more comprehensive metabolites to discover additional metabolites with causal associations. Secondly, the GWAS summary data of the gut microbiota we used was obtained through 16S rRNA gene sequencing, which is not precise at the species-level. Follow-up investigations can employ shotgun metagenomic sequencing analyses to obtain more specific and accurate data for inferring causal associations. Additionally, the GWAS summary data represent lifelong genetic exposure. Therefore, further clinical studies and animal experiments are needed to investigate whether the causal inferences derived from MR analysis can represent the short-term effects of gut microbiota changes on the host. Thirdly, although the heritability of CAD is independent of cognitive impairment and presents randomness (75), there may exist shared genetic foundations between CAD and cognitive impairment. This may result in missing SNPs that can be used as instrumental variables. Therefore, the results obtained by analyzing CAD and cognitive impairment separately may introduce some biases for the disease cluster. Subsequent studies should focus on researching the shared genetic basis of CAD and cognitive impairment to identify more precise gut microbiota and blood metabolites with causal associations. Fourthly, our study included only individuals of European descent, and caution should be exercised when extrapolating our results to other populations. Additionally, further research should be conducted on other ethnic groups.

In conclusion, this study investigated the causal associations between gut microbiota, blood metabolites, and the disease cluster of CAD combined with cognitive impairment. Using MR-BMA, we identified the blood metabolites and gut microbiota with the strongest causal associations. In addition, we further explored the mediating role of blood metabolites in the relationship between gut microbiota and disease clusters. Our study will provide valuable insights for the discovery of biomarkers as well as therapeutic targets for the disease cluster of CAD combined with cognitive impairment.

## Data availability statement

The original contributions presented in the study are included in the article/**Supplementary Material**. Further inquiries can be directed to the corresponding authors.

## Author contributions

SX: Formal Analysis, Methodology, Visualization, Writing – original draft. YFL: Data curation, Formal Analysis, Methodology, Software, Writing – original draft. QW: Data curation, Visualization, Writing – review & editing. FL: Formal Analysis, Resources, Writing – review & editing. YX: Investigation, Methodology, Writing – review & editing. FX: Data curation, Formal Analysis, Funding acquisition, Resources, Writing – review & editing. YL: Conceptualization, Funding acquisition, Investigation, Project administration, Writing – review & editing.
